# Supplementary material for: Heat-fueled enzymatic cascade for selective oxyfunctionalization of hydrocarbons
Source: Nat Commun. 2022 Jun 29;13:3741. doi: 10.1038/s41467-022-31363-8 (PMC9243031; doi:10.1038/s41467-022-31363-8)
Supplement: Supplementary file 1 — Supplementary Information [file 41467_2022_31363_MOESM1_ESM.pdf]

Supporting information

Heat-fueled enzymatic cascade for selective oxyfunctionalization of hydrocarbons

Jaeho Yoon[a], *Hanhwi Jang*[a], *Min-Wook Oh*[b], *Thomas Hilberath*[c], *Frank Hollmann*[c], *Yeon Sik Jung*[*,a], *and Chan Beum Park*[*,a]

[a] Department of Materials Science and Engineering, Korea Advanced Institute of Science and Technology (KAIST), 291 Daehak-ro, Daejeon 34141, Republic of Korea.
[b] Department of Materials Science and Engineering, Hanbat National University (HBNU), 125 Dongseodae-ro, Daejeon 34158, Republic of Korea.
[c] Department of Biotechnology, Delft University of Technology, Van der Maasweg 9, Delft 2629HZ, The Netherlands.
*Email: parkcb@kaist.ac.kr (C.B.P), ysjung@kaist.ac.kr (Y.S.J)

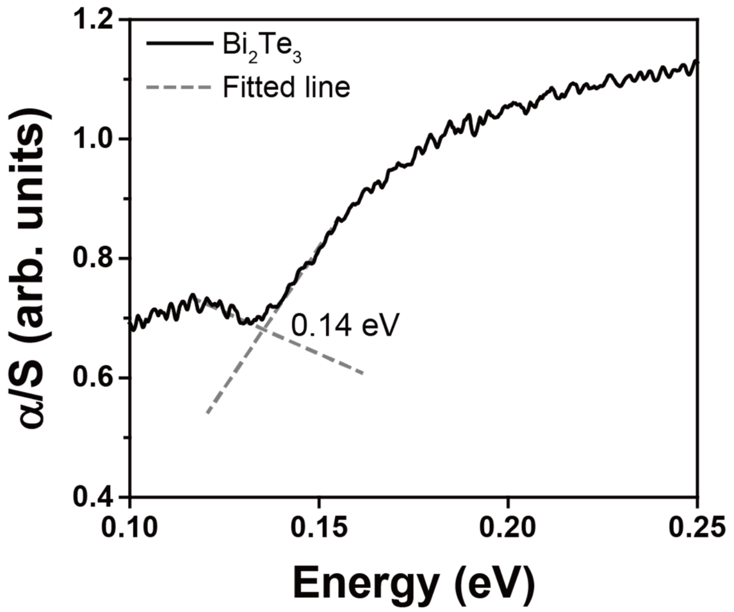

Supplementary Figure 1. Optical band gap measurement of the synthesized Bi_2Te_3 particles. The Kubelka-Munk function (α/S) was calculated from the diffuse-reflectance infrared spectroscopy (DRIFTS). The linear interpolation between free carrier absorption region (above 0.15 eV) and defect absorption region (below 0.12 eV) gives the optical band gap of 0.14 eV.

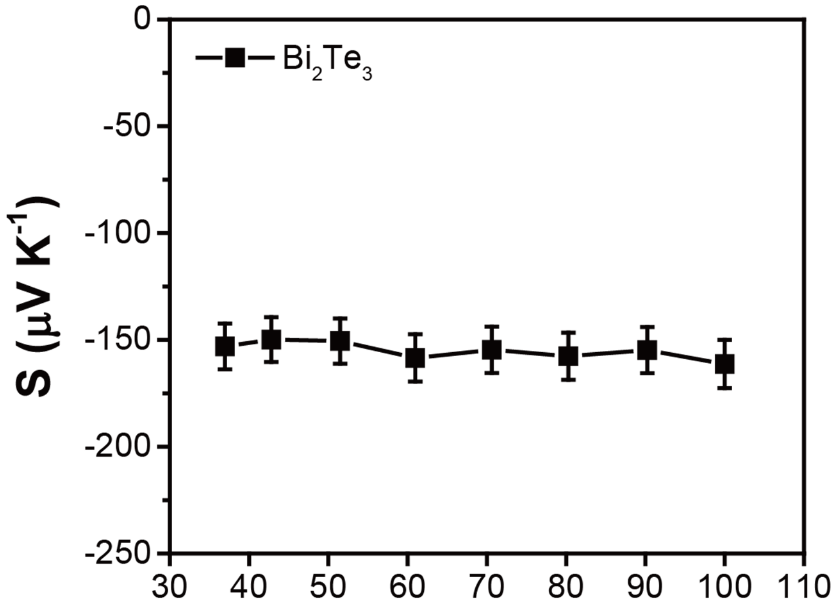

Supplementary Figure 2. Temperature-dependent absolute Seebeck coefficient of as-synthesized Bi_2Te_3. Error bar denotes standard error of Seebeck measurement, which is approximately 7%.

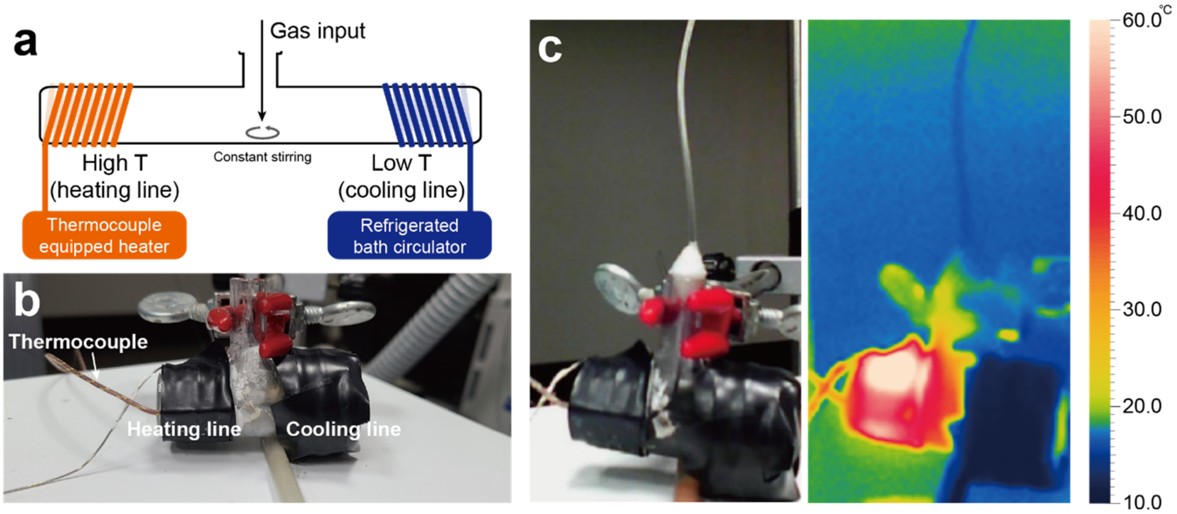

Supplementary Figure 3. a An illustration of thermoelectrobiocatalytic reactor setup with thermocouple equipped heater and refrigerated bath circulator. The reaction solution is continuously mixed using a magnetic stirrer with stirring speed of 200 rpm during reactions. **b** A digital photograph of the homebuilt reactor. The heater temperature was stabilized using PID controller. The thermocouple and heating wire were fixed to the reactor using a ceramic adhesive. **c** Digital photographs recorded by thermal imaging infrared camera during thermoelectrocatalytic reactions. The thermal image clearly shows the temperature gradient along the reactor.

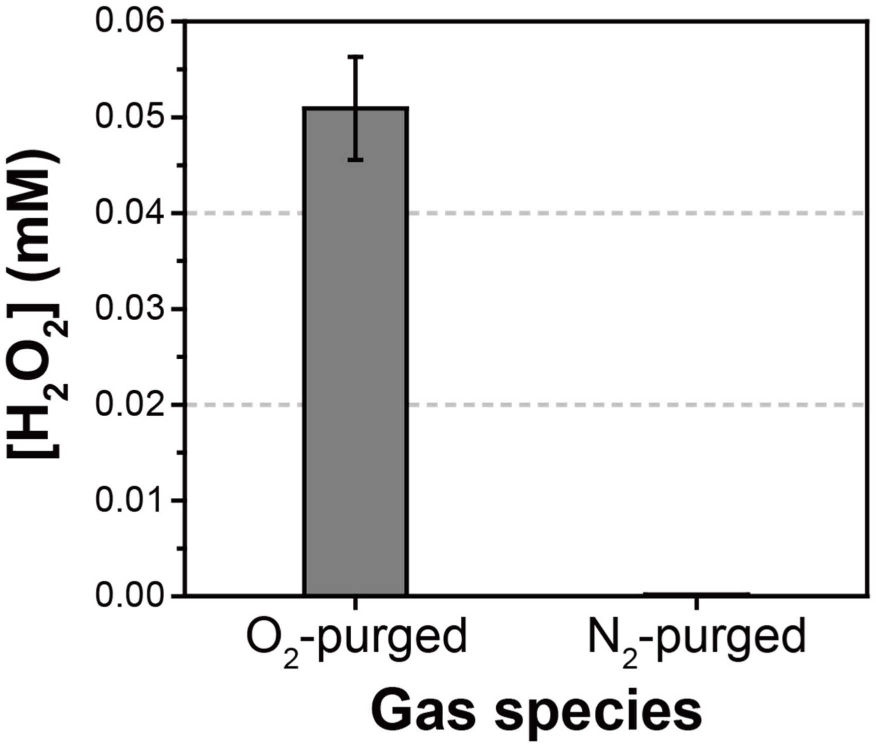

Supplementary Figure 4. Effect of ambient gas atmosphere on thermoelectrocatalytic generation of H_2O_2. Reaction conditions: 5 mg mL^{-1} Bi_2Te_3 dispersed in an O_2- or N_2-purged potassium phosphate-buffered solution (KPB, 100 mM, pH 7.0) with applied temperature difference (ΔT = 45 K) for 1 h. All reported values represent the mean ± standard deviation (n = 3).

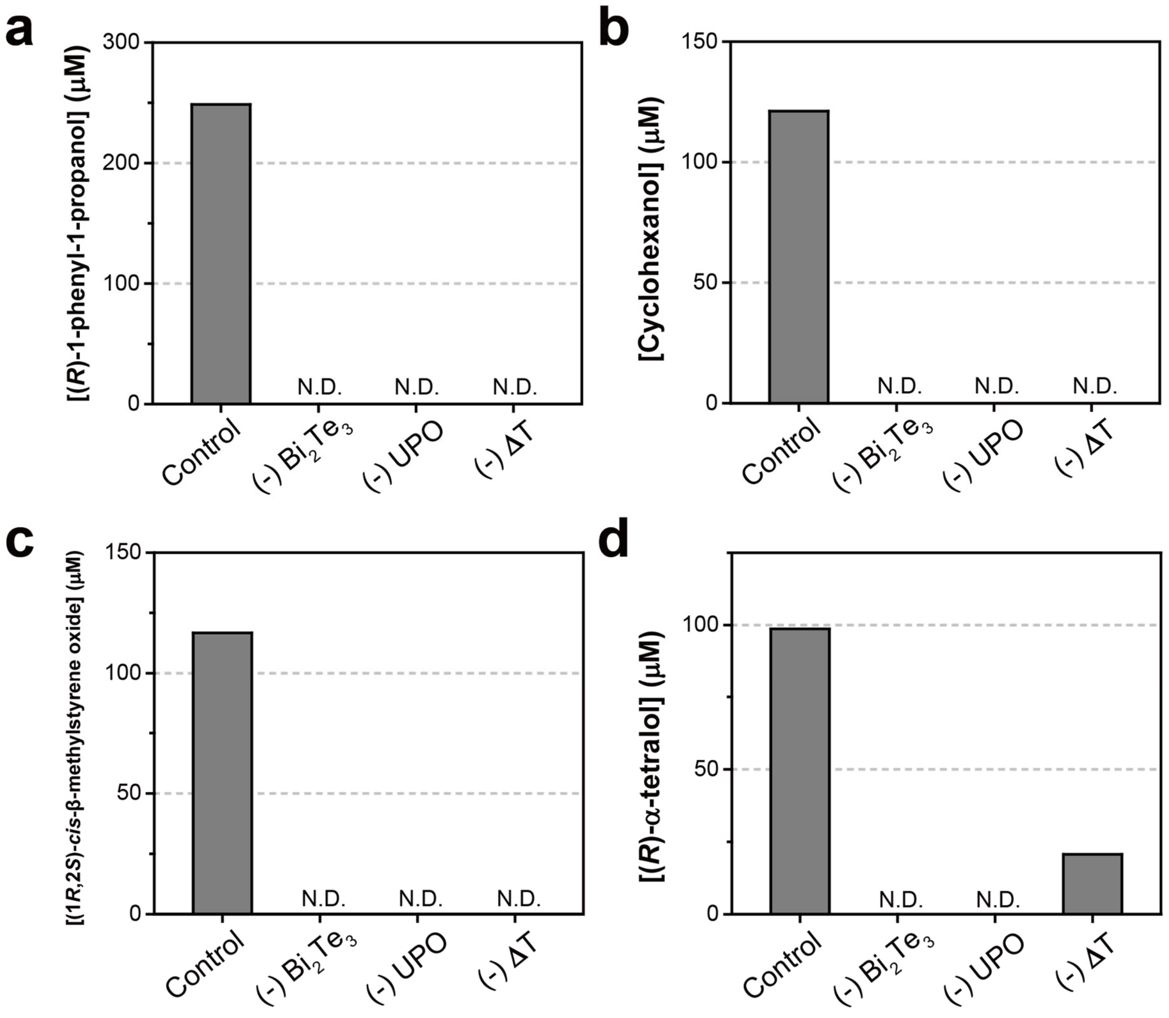

Supplementary Figure 5. A series of control experiments of each reaction component (i.e., Bi_2Te_3, r*Aae*UPO, and ΔT) on selected peroxygenase-catalyzed reactions [selective oxygenation of (**a**) propylbenzene; (**b**) cyclohexane; (**c**) *cis*-β-methylstyrene; and (**d**) tetralin]. Reaction conditions of positive control groups: 5 mg mL^{-1} Bi_2Te_3, 25 nM r*Aae*UPO, and 100 mM substrates dispersed in an O_2-purged KPB (100 mM, pH 7.0) with applied ΔT (45 K). For styrene epoxidation of *cis*-β-methylstyrene, we applied ΔT of 35 K to avoid the ignition of *cis*-β-methylstyrene. Reaction time = 2 h. The negative sign (-) denotes that the component is excluded as a control experiment. N.D. = not detected.

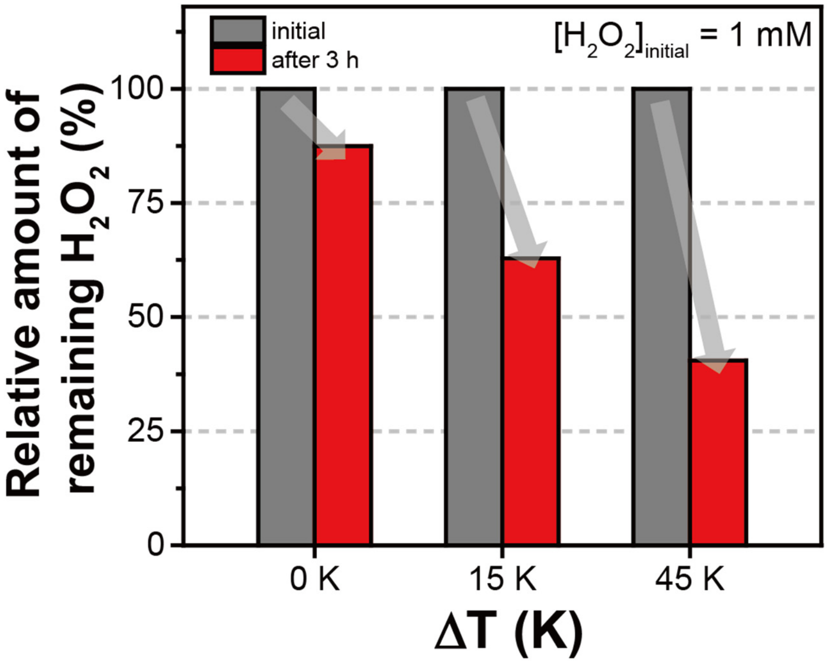

Supplementary Figure 6. Effect of the degree of absolute value of ΔT on decomposition of H_2O_2 for 3 h of reaction. Reaction conditions: 5 mg mL^{-1} Bi_2Te_3 dispersed in an O_2-purged KPB (100 mM, pH 7.0). Initial concentration of H_2O_2 in solution was 1 mM.

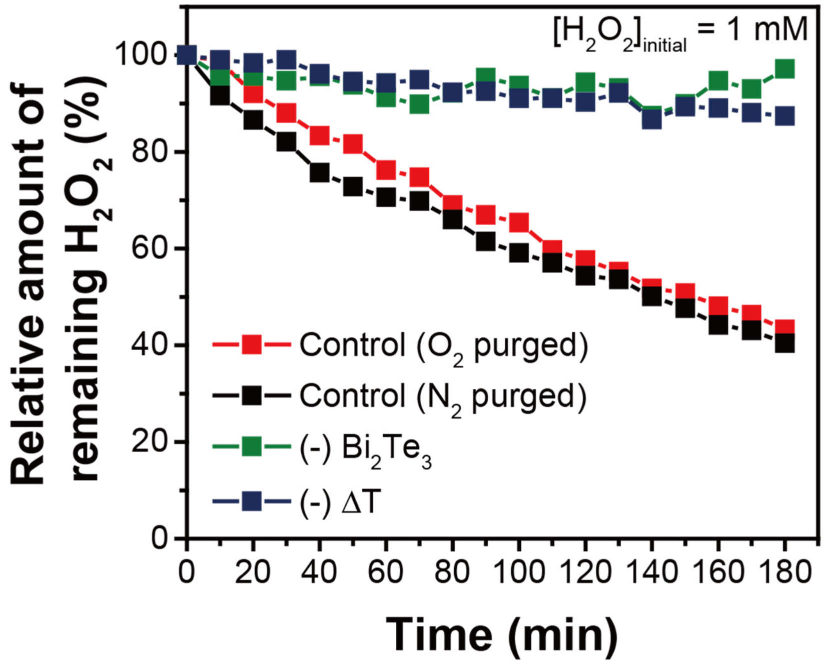

Supplementary Figure 7. Effect of the omission of each reaction component (i.e., Bi_2Te_3, ΔT) and ambient gas atmosphere on thermoelectrocatalytic decomposition of H_2O_2. Reaction conditions of control groups: 5 mg mL^{-1} Bi_2Te_3 dispersed in an O_2- or N_2-purged KPB (100 mM, pH 7.0) with applied ΔT (45 K). For experimental group without ΔT, the reaction solution was incubated at room temperature. Note that the initial concentration of H_2O_2 in solution was 1 mM. The negative sign (-) denotes that the component is excluded as a control experiment.

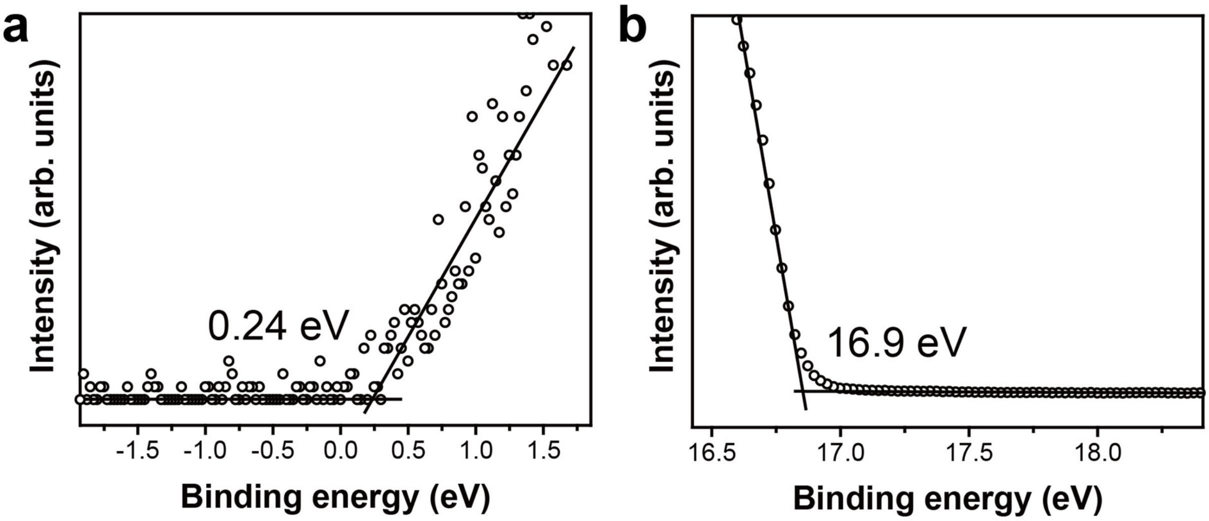

Supplementary Figure 8. Ultraviolet photoelectron spectroscopy (UPS) spectra of as-synthesized n-type Bi_2Te_3 for (**a**) the valence band and (**b**) the secondary electron cut-off region. He I radiation source having a photon energy of 21.2 eV was used for the measurement, yielding a Fermi level energy (E_F) of 4.3 eV with respect to the vacuum level.

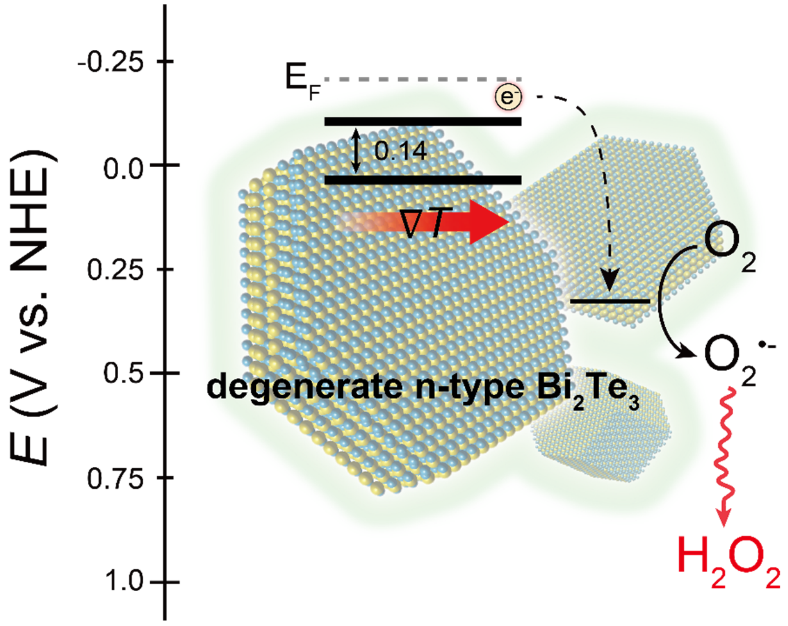

Supplementary Figure 9. Schematic energy diagram of as-synthesized n-type Bi_2Te_3 particles for thermoelectrocatalytic O_2 reduction to H_2O_2. The conduction band minimum potential and the electron chemical potential (i.e., the Fermi level, E_F) is more negative than the redox potential of $O_2/O_2^{\bullet-}$, thus, electrons can be transferred from the surface of Bi_2Te_3 to O_2 in the electrolyte, resulting the formation of H_2O_2. The energy diagram is determined from the DRIFTS and UPS measurements.

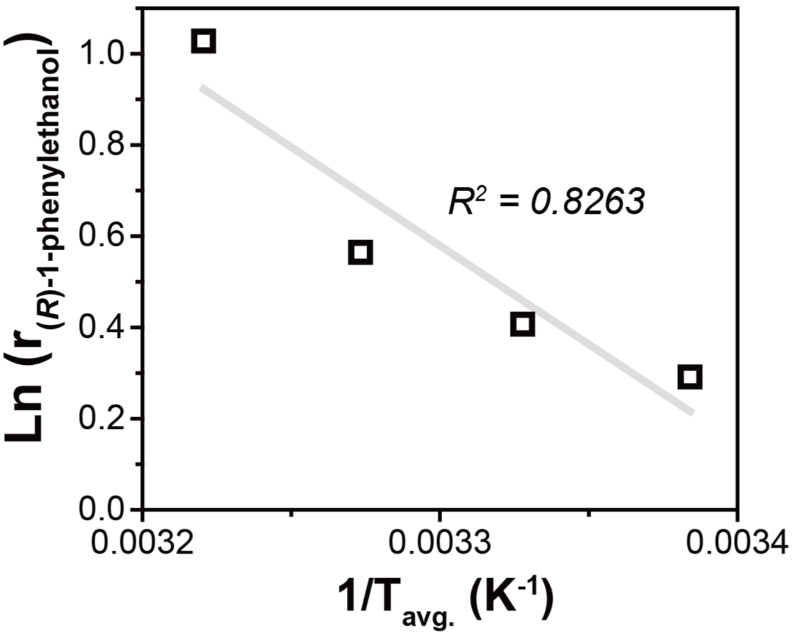

Supplementary Figure 10. Arrhenius relationship between average reaction temperature and ethylbenzene-to-(*R*)-1-phenylethanol conversion rate of UPOs. Reaction conditions: 5 mg mL^{-1} Bi_2Te_3, 25 nM r*Aae*UPO, and 100 mM ethylbenzene dispersed in an O_2-purged KPB (100 mM, pH 7.0) with applied ΔT.

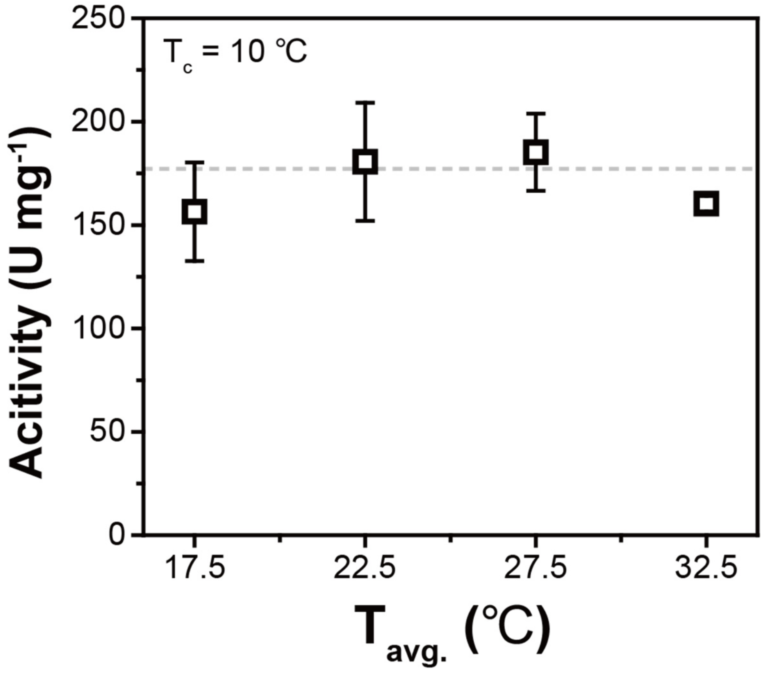

Supplementary Figure 11. Effect of average reaction temperature of the reactor on the activity of r*Aae*UPO. Enzyme activity was measured after 1 h of incubation at given conditions. A white gray dashed line represents the average initial activity of r*Aae*UPO (176.92 U mg^{-1}). Reaction conditions: 200 nM r*Aae*UPO dispersed in a KPB (100 mM, pH 7.0) with applied ΔT. Temperature at cool side (T_c) was fixed to 10 °C and hot-side temperature was carefully controlled using PID controller. All reported values represent the mean ± standard deviation (n = 3).

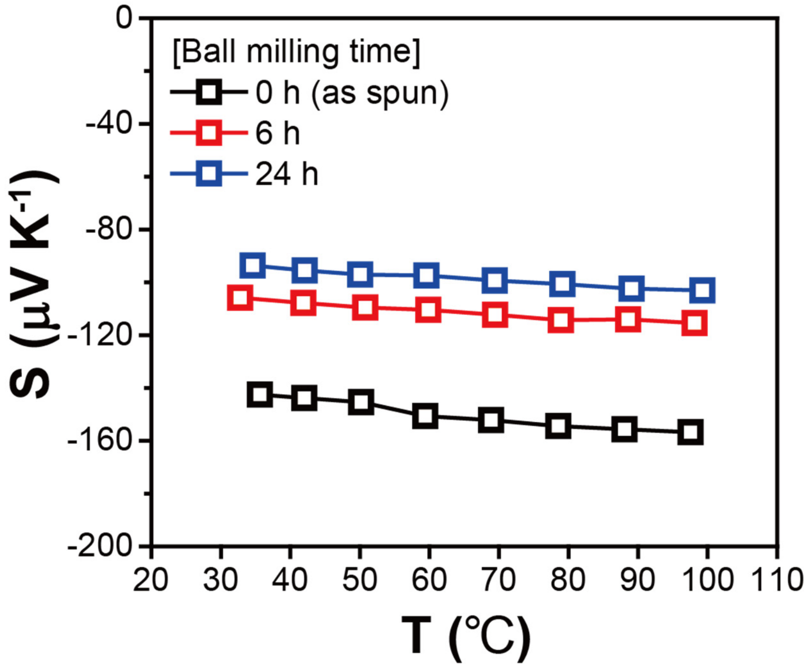

Supplementary Figure 12. Temperature-dependent absolute Seebeck coefficient of high-energy ball milled Bi_2Te_3 particles. The linear increase of |*S*| with increasing temperature confirms that all prepared samples are degenerate semiconductors.

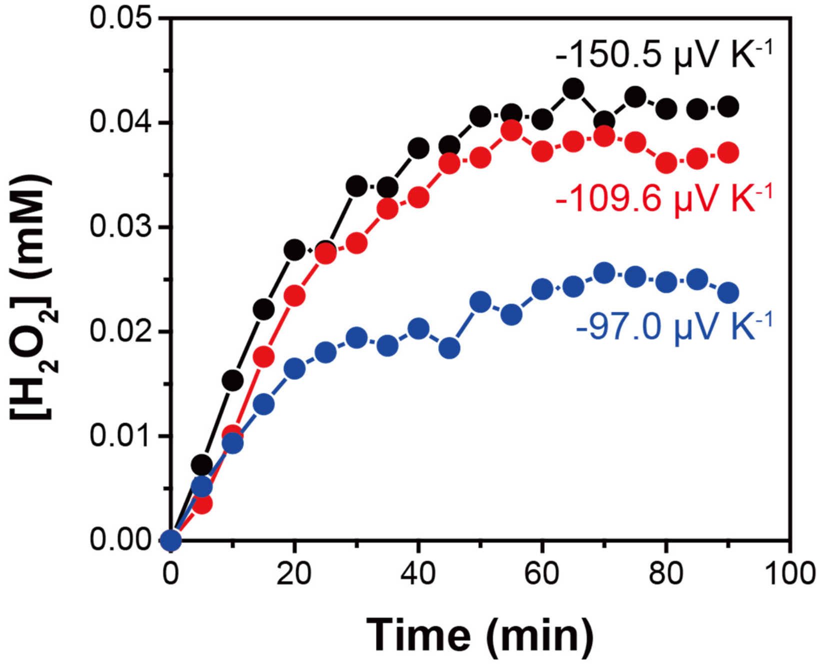

Supplementary Figure 13. A time course of thermoelectrocatalytic H_2O_2 generation by the high-energy ball milled Bi_2Te_3 particles with different S under O_2 atmosphere for 90 min of reaction. Reaction conditions: 5 mg mL^{-1} Bi_2Te_3 dispersed in KPB (100 mM, pH 7.0) with applied ΔT (45 K).

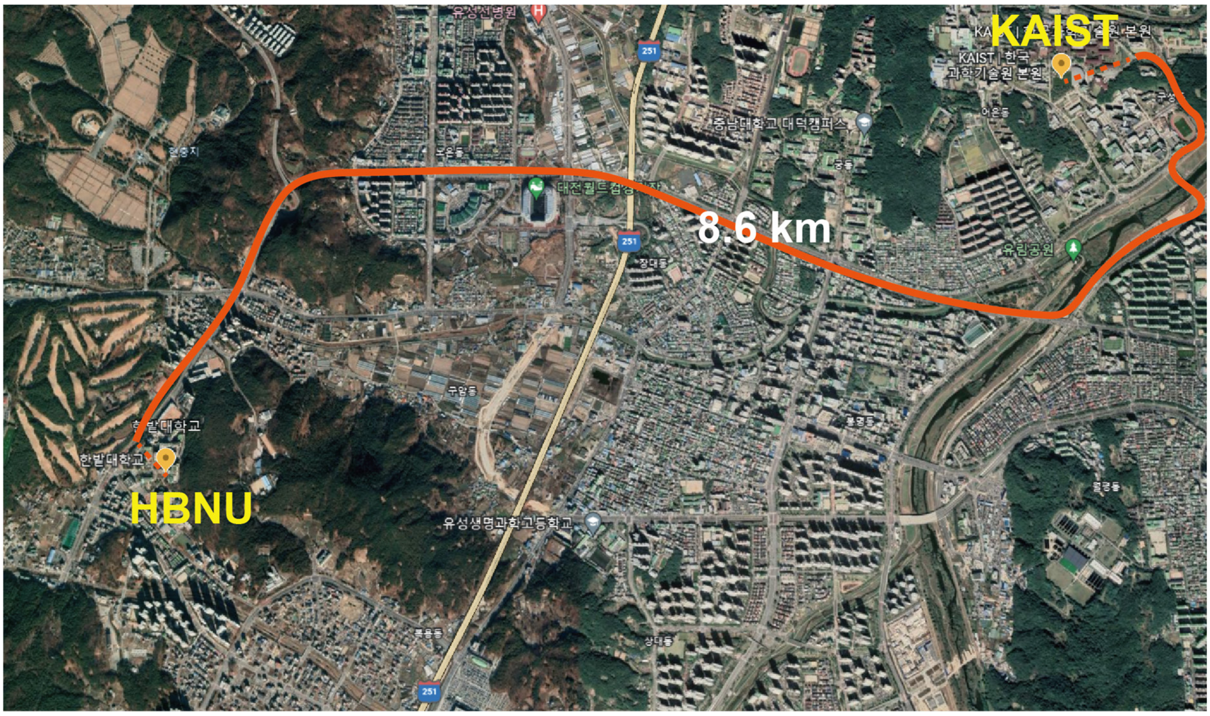

Supplementary Figure 14. A satellite map (Map data: ©2022 Google, Google Earth) of one way route from Korea Advanced Institute of Science and Technology (KAIST) to Hanbat National University (HBNU). Distance of this route is approximately 8.6 km.

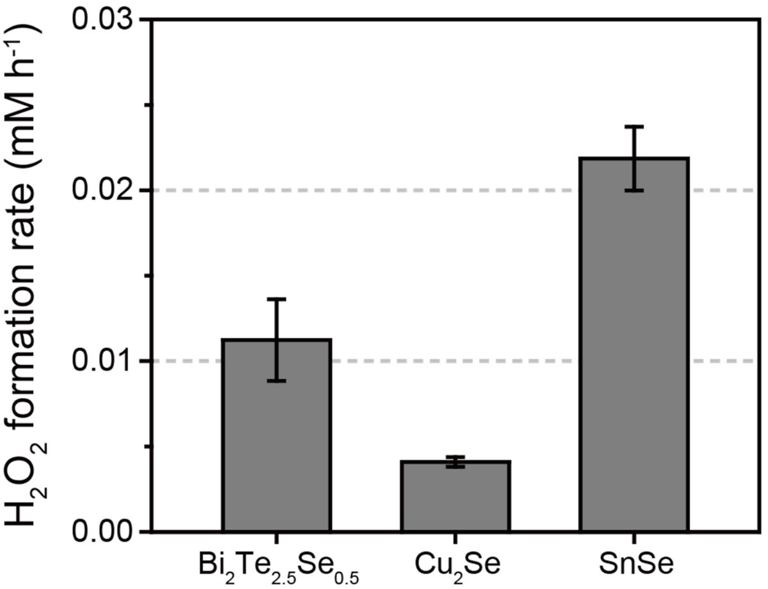

Supplementary Figure 15. Rate of H_2O_2 generation of other representative thermoelectric materials. Reaction conditions: 5 mg mL^{-1} of $Bi_2Te_{2.5}Se_{0.5}$, 10 mg mL^{-1} of Cu_2Se, or 10 mg mL^{-1} of SnSe dispersed in an O_2-purged KPB (100 mM, pH 7.0) with applied ΔT (45 K). Note that $Bi_2Te_{2.5}Se_{0.5}$ is n-type, while Cu_2Se and SnSe are p-type semiconductors.

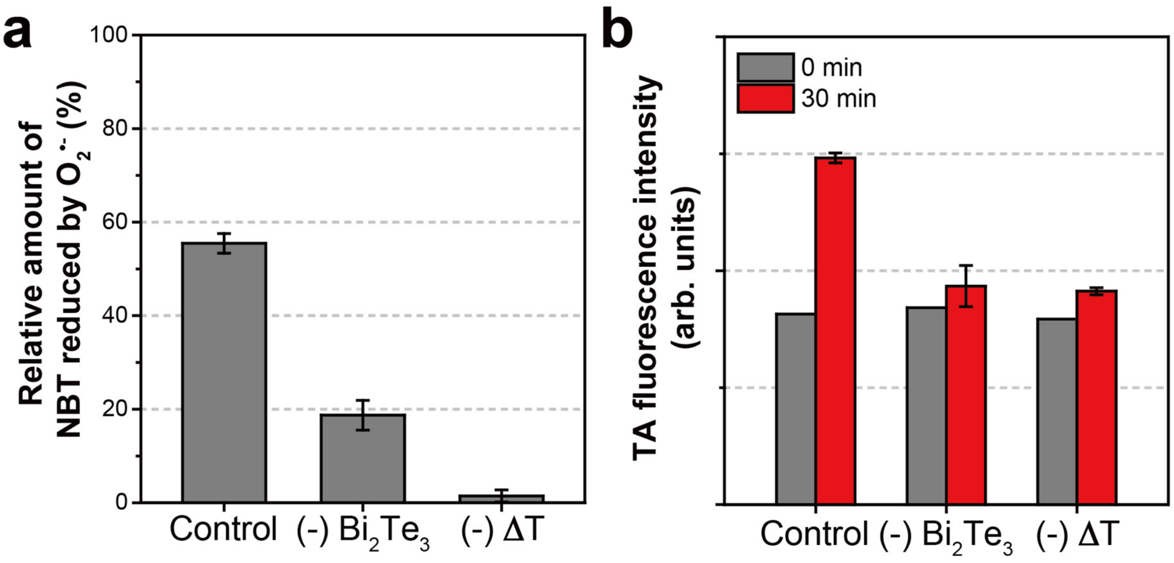

Supplementary Figure 16. Qualitative analyses of $O_2^{\bullet-}$ and $OH^{\bullet}$ generation during thermoelectrocatalytic H_2O_2 generation via a series of (**a**) nitro blue tetrazolium (NBT, 20 μM) and (**b**) terephthalic acid (TA, 300 μM) assays with the omission of Bi_2Te_3 (5 mg mL^{-1}) and ΔT (45 K), respectively. Solvent: an O_2-purged KPB (100 mM, pH 7.0). Reaction time: 1 h for NBT assays and 30 min for TA assays. All reported values represent the mean ± standard deviation (n = 3). The negative sign (-) denotes that the component is excluded as a control experiment.

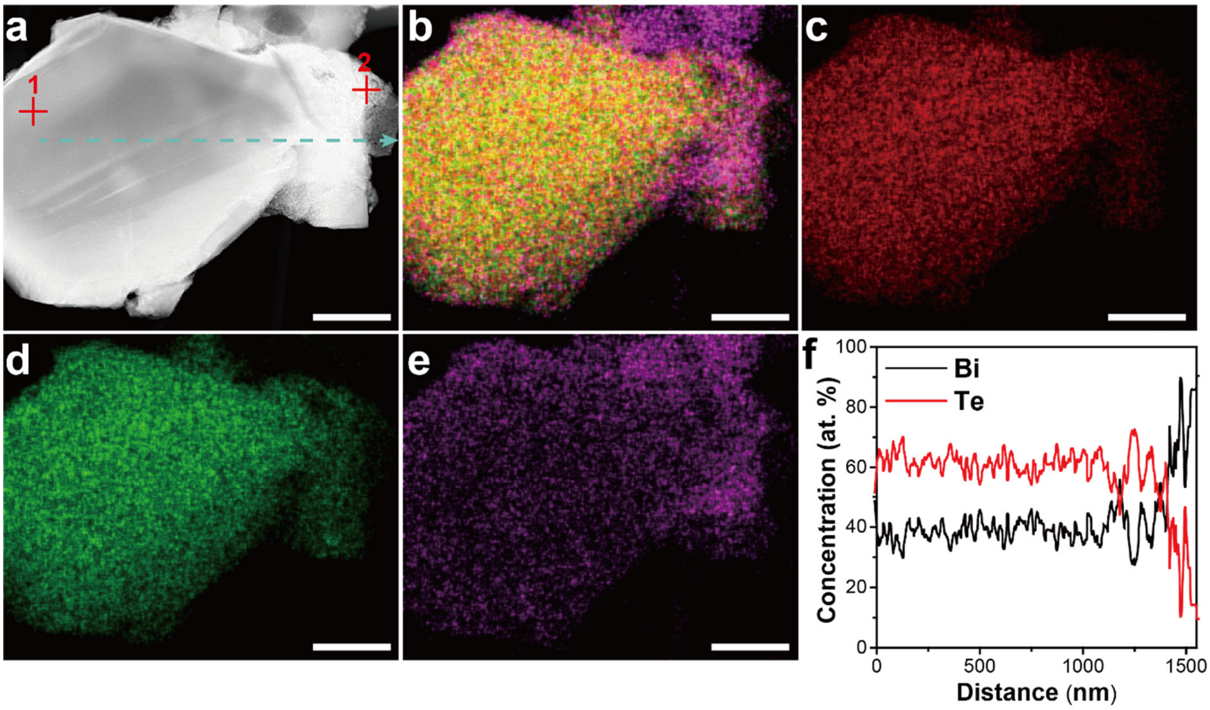

Supplementary Figure 17. a HAADF-STEM image of Bi_2Te_3 particles after 8 h of thermoelectrobiocatalytic reactions. Representative EDS elemental mapping images of (**b**) overlay, (**c**) Bi, (**d**) Te, and (**e**) O. Scale bar = 300 nm. **f** STEM-EDS quantification results of dashed line in (**a**) showing an abrupt loss of Te due to oxygen incorporation during the reaction.

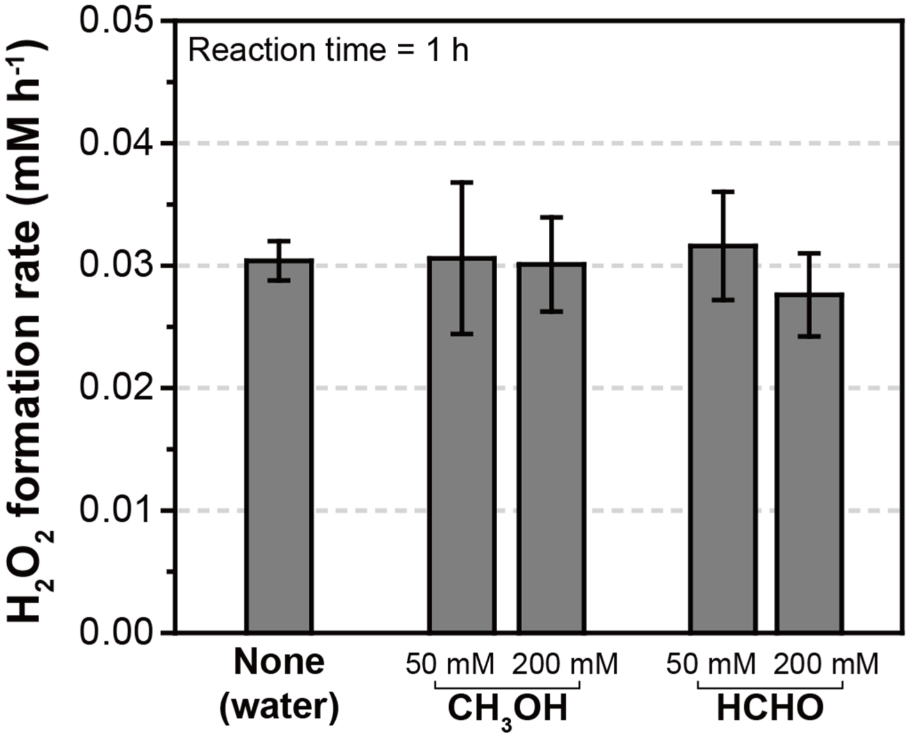

Supplementary Figure 18. Effect of typical electron donors on Bi_2Te_3-driven thermoelectrocatalytic generation of H_2O_2. Reaction conditions: 5 mg mL^{-1} Bi_2Te_3 dispersed in an O_2-purged potassium phosphate-buffered solution (KPB, 100 mM, pH 7.0) with applied temperature difference (ΔT = 45 K) for 1 h. All reported values represent the mean ± standard deviation (n = 3).

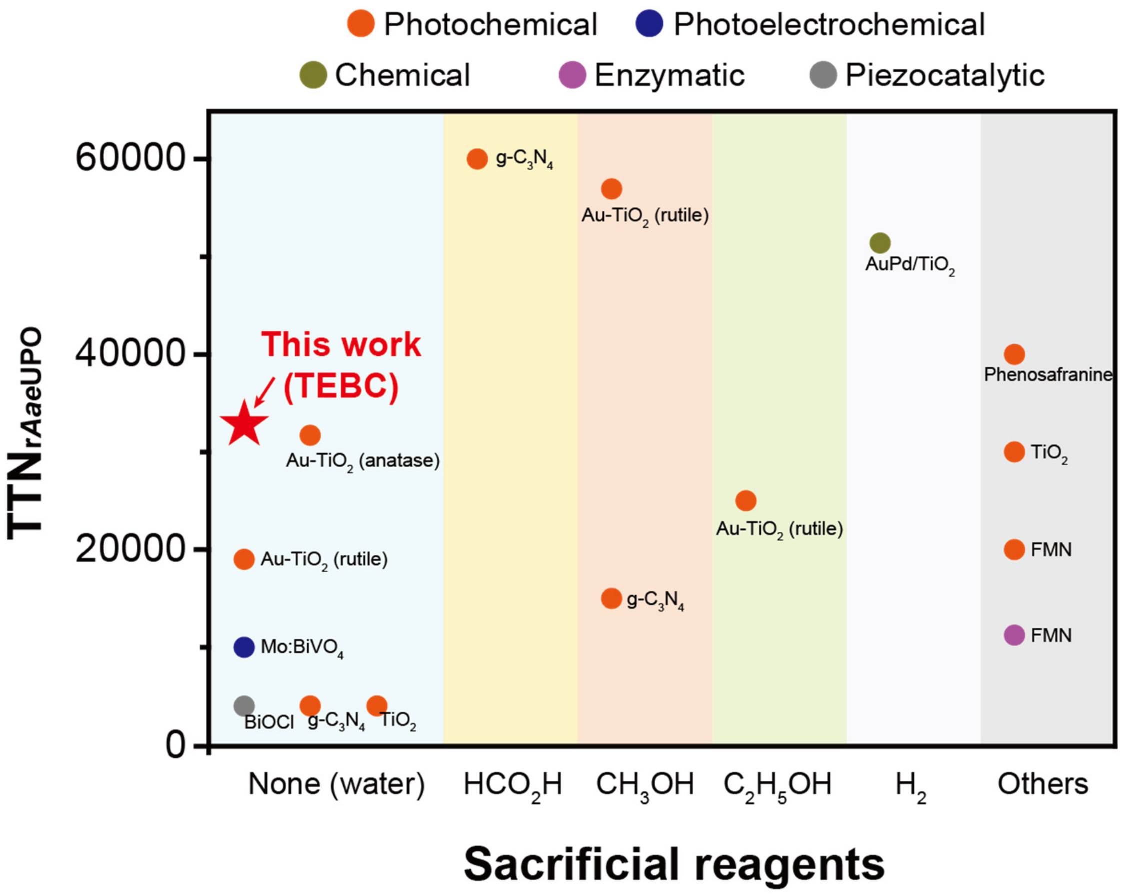

Supplementary Figure 19. Comparison of total turnover number of r*Aae*UPO ($TTN_{rAaeUPO}$) values achieved for UPO-catalyzed selective oxyfunctionalization reactions in this study and up-to-date r*Aae*UPO-driven biocatalytic systems. The catalysts include gold-loaded TiO_2 (Au-TiO_2)[1, 2], Mo-doped $BiVO_4$ (Mo:$BiVO_4$)[3], BiOCl[4], graphitic carbon nitride (g-C_3N_4)[5], TiO_2[6], AuPd decorated TiO_2 (AuPd/TiO_2)[7], phenosafranine[8], flavin mononucleotide (FMN).[8, 9]

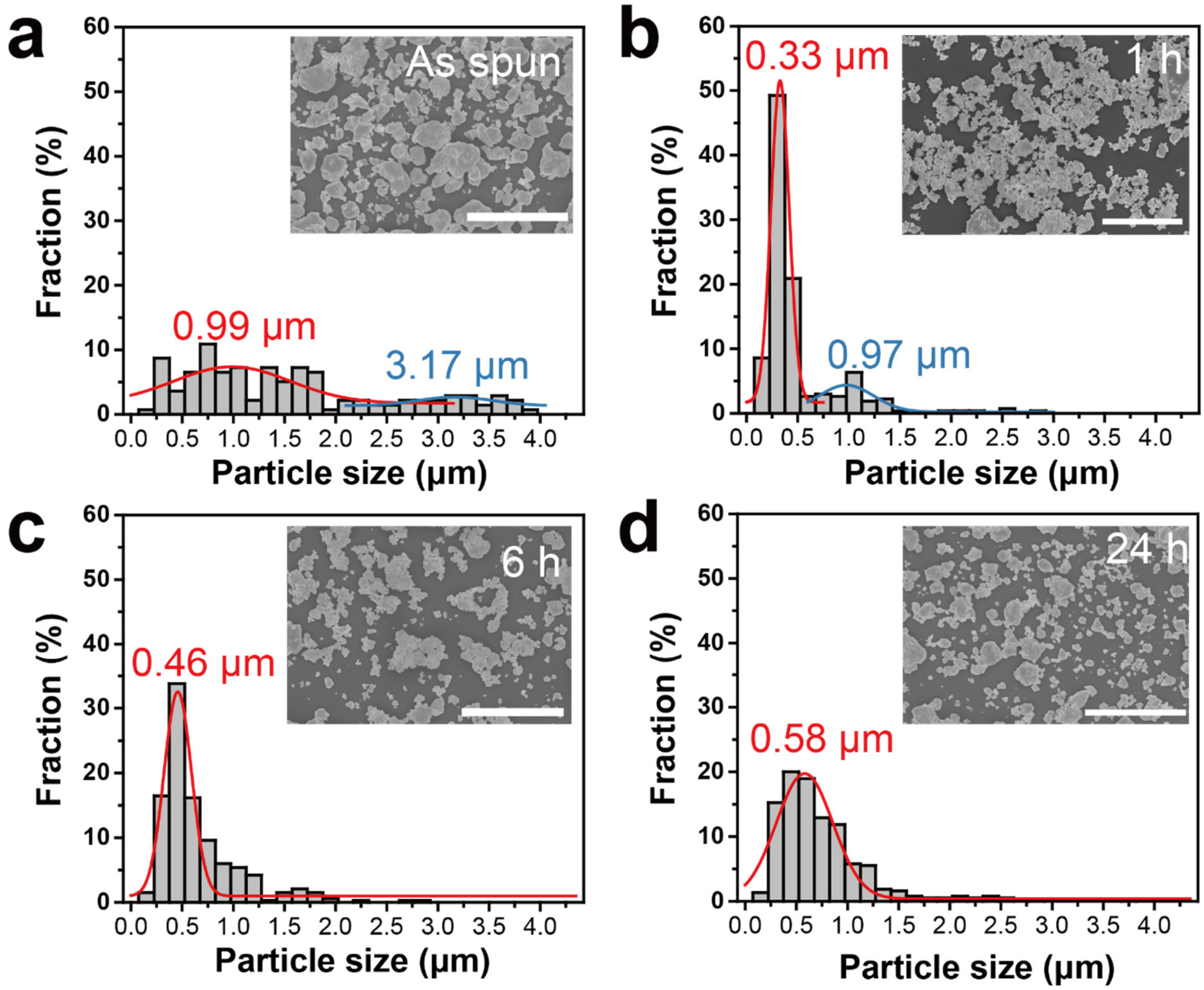

Supplementary Figure 20. Effect of ball milling time on size distribution of Bi_2Te_3 particles. Measured spatial size distribution and corresponding Gaussian distribution fitting of Bi_2Te_3 particles showed that high-energy ball milling significantly reduces the particle size of Bi_2Te_3. Insets are SEM images of Bi_2Te_3 powders at given milling time. Scale bar = 10 μm. Total counts for each sample [(**a**) as spun, (**b**) 1 h, (**c**) 6 h, and (**d**) 24 h) are 138, 268, 334, and 380, respectively. Rotational speed of ball milling process = 500 rpm.

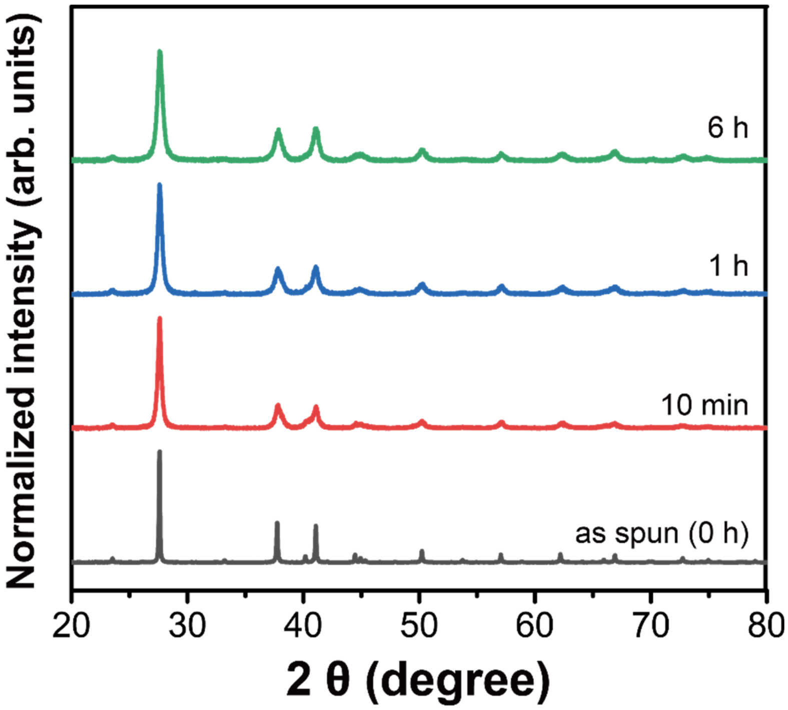

Supplementary Figure 21. Normalized powder XRD pattern of as spun and high-energy ball-milled Bi_2Te_3 samples (10 min, 1 h, 6 h). Crystallinity of synthesized Bi_2Te_3 particles decreases with increasing milling time. Intensities were normalized to [0, 1]. Rotational speed of ball milling process = 500 rpm.

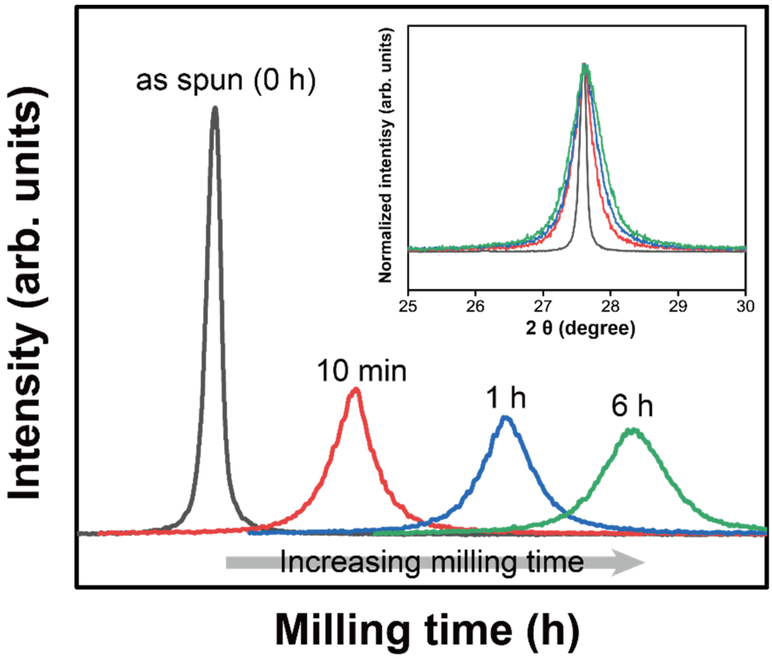

Supplementary Figure 22. The powder XRD peaks of (0015) plane for as spun and high-energy ball-milled samples (10 min, 1 h, 6 h). The decrease of the peak intensity as well as a peak broadening (inset) show a decrease of crystallite size and amorphization of Bi_2Te_3 with increasing milling time. Rotational speed of ball milling process = 500 rpm.

Supplementary Table 1. Charge transport properties of Bi_2Te_3 at room temperature.

Sample name	Conduction type	Seebeck coefficient ($\mu V \cdot K^{-1}$)	Hall carrier concentration (cm^{-3})	Band effective mass (m_0)
Bi_2Te_3	N-type	-153.062	3.04×10^{19}	0.7

Supplementary Table 2. GC oven temperature programs and retention times for substrates and products

Substrate	Temperature profile	Retention time (min)
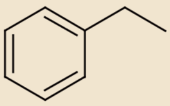<br>Ethylbenzene	90 °C for 5 min, 20 °C/min to 110 °C and hold for 13.5 min, 40 °C/min to 180 °C and hold for 1.5 min	Ethylbenzene: 4.3<br>Acetophenone: 9.3<br>(*R*)-1-Phenylethanol: 15.4<br>(*S*)-1-Phenylethanol: 16.5<br>1-Octanol (internal standard): 11.0
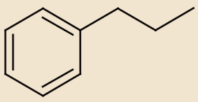<br>Propylbenzene	90 °C for 5 min, 20 °C/min to 130 °C and hold for 10 min	Propylbenzene: 5.8<br>(*R*)-1-Phenyl-1-propanol: 12.7<br>(*S*)-1-Phenyl-1-propanol: 13.4<br>Propiophenone: 10.0<br>1-Octanol (internal standard): 9.0
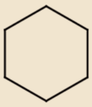<br>Cyclohexane	90 °C for 5 min, 20 °C/min to 110 °C and hold for 5 min, 40 °C/min to 150 °C and hold for 3 min	Cyclohexane: 2.9<br>Cyclohexanone: 6.0<br>Cyclohexanol: 8.1<br>1-Octanol (internal standard): 10.1
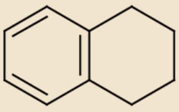<br>Tetralin (1,2,3,4-Tetrahydronaphthalene)	90 °C for 1 min, 5 °C/min to 140 °C and hold for 10 min, 80 °C/min to 180 °C and hold for 1 min	Tetralin: 9.0<br>(*R*)-α-Tetralol: 20.14<br>(*S*)-α-Tetralol: 19.78<br>α-Tetralone: 15.934<br>1-Octanol (internal standard): 8.46
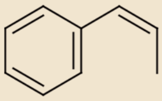<br>*cis*-β-methylstyrene	90 °C for 2 min, 20 °C/min to 150 °C and hold for 7 min, 20 °C/min to 180 °C and hold for 1 min	*cis*-β-Methylstyrene: 4.75<br>(1*R*,2*S*)-*cis*-β-Methylstyrene oxide [a]: 6.23<br>(1*S*,2*R*)-*cis*-β-Methylstyrene oxide: 6.09

[a] The concentration of (1*R*,2*S*)-*cis*-β-methylstyrene oxide) was calculated using (1*S*,2*R*)-*cis*-β-methylstyrene oxide as an internal standard.

Supplementary References

(1) Zhang, W.; Burek, B. O.; Fernandez-Fueyo, E.; Alcalde, M.; ́Bloh, J. Z.; Hollmann, F. Selective Activation of C-H Bonds in a Cascade Process Combining Photochemistry and Biocatalysis. *Angew. Chem. Int. Ed*. **2017**, *56*, 15451−15455.

(2) Zhang, W.; Fernández-Fueyo, E.; Ni, Y.; van Schie, M. M. C. H.; Gacs, J.; Renirie, R.; Wever, R.; Mutti, F. G.; Rother, D.; Alcalde, M.; Hollmann, F. Selective aerobic oxidation reactions using a combination of photocatalytic water oxidation and enzymatic oxyfunctionalizations. *Nat. Catal.* **2018**, *10*, 55-62.

(3) Choi, D. S.; Kim, J.; Hollmann, F.; Park, C. B. Solar-Assisted eBiorefinery: Photoelectrochemical Pairing of Oxyfunctionalization and Hydrogenation Reactions. *Angew. Chem. Int. Ed.* **2020**, *59*, 15886-15890.

(4) Yoon, J.; Kim, J.; Tieves, F.; Zhang, W.; Alcalde, M.; Hollmann, F.; Park, C. B. Piezobiocatalysis: Ultrasound-Driven Enzymatic Oxyfunctionalization of C-H Bonds. *ACS Catal.* **2020**, *10*, 5236-5242.

(5) van Schie, M. M. C. H.; Zhang, W.; Tieves, F.; Choi, D. S.; Park, C. B.; Burek, B. O.; Bloh, J. Z.; Arends, I. W. C. E.; Paul, C. E.; Alcalde, M.; Hollmann, F. Cascading g-C_3N_4 and Peroxygenases for Selective Oxyfunctionalization Reactions. *ACS Catal*. **2019**, *9*, 7409−7417.

(6) Burek, B. O.; de Boer, S. R.; Tieves, F.; Zhang, W.; van Schie, M. M. C. H.; Bormann, S.; Alcalde, M.; Holtmann, D.; Hollmann, F.; Bahnemann, D. W.; Bloh, J. Z. Photoenzymatic Hydroxylation of Ethylbenzene Catalyzed by Unspecific Peroxygenase: Origin of Enzyme Inactivation and the Impact of Light Intensity and Temperature. *ChemCatChem* **2019**, *11*, 3093-3100.

(7) Freakley, S. J.; Kochius, S.; van Marwijk, J.; Fenner, C.; Lewis, R. J.; Baldenius, K.; Marais, S. S.; Opperman, D. J.; Harrison, S. T. L.; Alcalde, M.; Smit, M. S.; Hutchings, G. J. A chemo-enzymatic oxidation cascade to activate C–H bonds with in situ generated H_2O_2. *Nat. Commun*. **2019**, *10*:4178.

(8) Willot, S. J. P.; Fernández-Fueyo, E.; Tieves, F.; Pesic, M.; Alcalde, M.; Arends, I. W. C. E.; Park, C. B.; Hollmann, F. Expanding the Spectrum of Light-Driven Peroxygenase Reactions. *ACS Catal.* **2019**, *9*, 890-894.

(9) Al-Shameri, A.; Willot, S. J. P; Paul, C. E.; Hollmann, F.; Lauterbach, L. H_2 as a fuel for flavin- and H_2O_2-dependent biocatalytic reactions. *ChemComm.* **2020**, *56*, 9667-9670.
